# Ocular rhinosporidiosis mimicking conjunctival squamous papilloma in Kenya – a case report

**DOI:** 10.1186/1471-2415-14-45

**Published:** 2014-04-08

**Authors:** Stephen Gichuhi, Timothy Onyuma, Ephantus Macharia, Joy Kabiru, Alain M’bongo Zindamoyen, Mandeep S Sagoo, Matthew J Burton

**Affiliations:** 1 Department of Ophthalmology, University of Nairobi, Nairobi, Kenya; 2 London School of Hygiene and Tropical Medicine, London, UK; 3 Department of Pathology, MP Shah Hospital, Nairobi, Kenya; 4 PCEA Kikuyu Hospital Eye Unit, Kikuyu, Kenya; 5 Moorfields Eye Hospital, London, UK; 6 UCL Institute of Ophthalmology, University College London, London, UK

**Keywords:** Ocular rhinosporidiosis, Rhinosporidium seeberi, Conjunctival papilloma, Toluidine-blue, Africa

## Abstract

**Background:**

Ocular rhinosporidiosis is a chronic granulomatous infection caused by a newly classified organism that is neither a fungus nor bacterium. It often presents as a benign conjunctival tumour but may mimic other ocular conditions. It is most often described in India. In Africa cases have been reported from South Africa, Kenya, Tanzania, Malawi, Uganda, Congo and Ivory Coast.

**Case presentation:**

A 54 year old man was seen in Kenya with a lesion that resembled a conjunctival papilloma. We report resemblance to conjunctival papilloma and the result of vital staining with 0.05% Toluidine Blue.

**Conclusion:**

Ocular rhinosporidiosis occurs in East Africa. It may resemble conjunctival squamous papilloma. Vital staining with 0.05% Toluidine blue dye did not distinguish the two lesions well.

## Background

Rhinosporidiosis is a chronic granulomatous infection of the mucous membranes (nasal, oral, ocular and rectal) caused by *Rhinosporidium seeberi*[1]. This is an unusual unicellular pathogen that is difficult to culture and whose taxonomic classification has been controversial. It has been variously hypothesized to be a cyanobacterium (prokaryote), a eukaryotic Mesomycetozoa or a fungus [2]. Currently it is domiciled in the Mesomycetozoea class (also known as the DRIP clade, or Ichthyosporea). The term Mesomycetozoea derives from “Meso-” (in the middle of), “-myceto-” (fungi) and “-zoea” (animals). This is a heterogeneous group of microorganisms at the animal-fungal boundary. The Mesomycetozoea are a small group of protists, which are mostly parasites of fish and other animals.

Rhinosporidiosis affects both adults and children and is commonly seen in otolaryngology. The largest reported case series of rhinosporidiosis consisting of 462 cases in India found that the disease mainly occurs in the nose and nasopharynx (81.1%) while the eye was affected in 14.2% [3]. Another series of 34 cases from India also found nasal and nasopharyngeal involvement in 85% while the eye was affected in 9% of cases [4]. A case involving multiple parts of the body; the nares, multiple areas of the skin, the external urethral meatus, glans of penis and the perineum has been reported in India [5].

Ocular rhinosporidiosis affecting the conjunctiva was first described in India in 1912 [6]. Currently most published reports on rhinosporidiosis of the eye have been reported from Asia mainly from India, Sri Lanka, Nepal and Bangladesh. In Africa it has been reported in South Africa, Malawi, Zambia, Kenya, Uganda, Tanzania, Congo, Ivory Coast, and Cameroon [7-13]. None of the cases in Africa were initially diagnosed as ocular rhinosporidiosis, perhaps a sign of its rarity.

How the disease is acquired remains an enigma. Rhinosporidiosis has been associated with migrants from endemic areas [14-16]. Although it is an infectious disease, as lesions are always associated with the presence of the pathogen, there is limited data on how it might be transmitted [15]. It is presumed to be acquired through traumatized nasal mucosa and spread to other sites by autoinoculation. As most rhinosporidiosis lesions arise from the nose, it is feasible that ocular involvement may occur by spreading from the nose through the lacrimal sac to the plica of the conjunctiva. This hypothesis is however unproven.

Ocular rhinosporidium most often presents as a polypoid mass of the palpebral conjunctiva [17]. It may also present as a lacrimal sac diverticulum [18], recurrent chalazion [19], conjunctival cyst [20], chronic follicular conjunctivitis in contact lens wearers [21], peripheral keratitis [22], scleral melting [23], ciliary staphyloma [24] or simulate a tumour of the eyelid [25] or periorbital skin [26]. Large conjunctival lesions may cause mechanical ectropion [27]. Lacrimal sac disease may present with bloody tears [16].

In a series of 63 cases from India that included nasal, nasopharyngeal and ocular disease, routine haematology tests did not show any abnormality and while cytology of smears obtained via fine needle aspiration or cytology has a role in diagnosis, the mainstay remains histology [28]. Vital staining with Toluidine blue has been described for diagnosis of conjunctival tumours but not for rhinosporidiosis [29].

The treatment is surgical excision with or without cautery at the base and recurrence is described as rare [30]. Scleral melting may be treated with a tectonic corneal graft [31]. There are reports that the following agents are effective in vivo: imidocarb diproprionate, diminazine aceturate, cycloserine, dapsone, trimethoprim-suphadiazine, ketoconazole, sodium stibogluconate, and amphotericin B [32]. Dapsone is the most commonly reported drug and combination therapy to prevent drug resistance is recommended. A laboratory study in India found that biocides including hydrogen peroxide, glutaraldehyde, chloroxylenol, chlorhexidine, cetrimide, thimerosal, 70% ethanol, iodine in 70% ethanol, 10% formalin, povidone-iodine, sodium azide and silver nitrate caused metabolic inactivation with or without altered structural integrity of the endoconidia of *Rhinosporidium seeberii* but no human trials have been reported [33]. Human anti-rhinosporidial antibody is not directly protective against the endoconidia [34].

## Case presentation

A 54-year-old male presented to the eye clinic at the PCEA Kikuyu Hospital on the outskirts of Nairobi with a 16 month history of a painless lump on the surface of the right eye. Concerned about the appearance, he attributed the lesion to a foreign body that entered that eye while he was trimming a hedge.

No other family member or neighbour had a similar disease. Social history included living in Homa Bay district on the shores of Lake Victoria from birth to 18 years age, then Kapsabet, a highland area in the Rift Valley until the age of 26 years, followed by Nairobi. He had resided in a low-income area of Nairobi for the past 11 years. Occupational history included working as a gardener for the last 10 years and a cook for 5 years prior to that. Although he grew up in a lakeside area, he had not dived or swum in stagnant water in the recent past.

**Figure 1:**
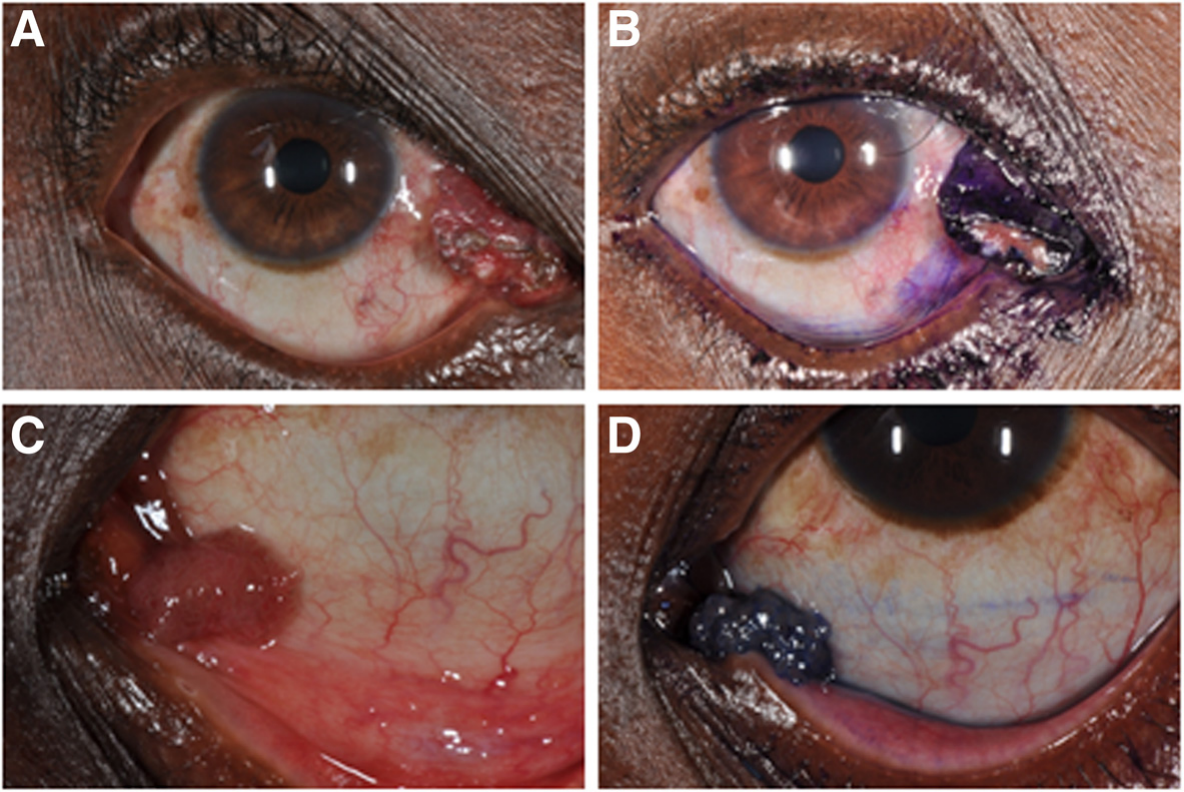
**Pre-operative clinical photographs. A** &**B** – shows the ocular rhinosporidiosis lesion in the region of the right medial canthus. **C** &**D** - shows squamous papilloma in the left medial canthus area from another patient for comparison. Both lesions are shown before and after vital staining with 0.05% Toluidine Blue.

On examination he had a pedunculated 6×11 mm wide fleshy mass at the medial canthus of the right eye (Figure 1), which was pink with some intrinsic pigmentation. It had a papilliform surface with vascular tufts and some epithelial ulceration. There was no discharge or conjunctival injection. The mass was not attached to the lid but arose from the plica semilunaris. On vital staining with 0.05% Toluidine Blue it was coloured deep blue except at the ulcerated surface, similar to the staining of a papilloma. The clinical diagnosis was of conjunctival papilloma and surgical excision under local anaesthetic was undertaken.

**Figure 2:**
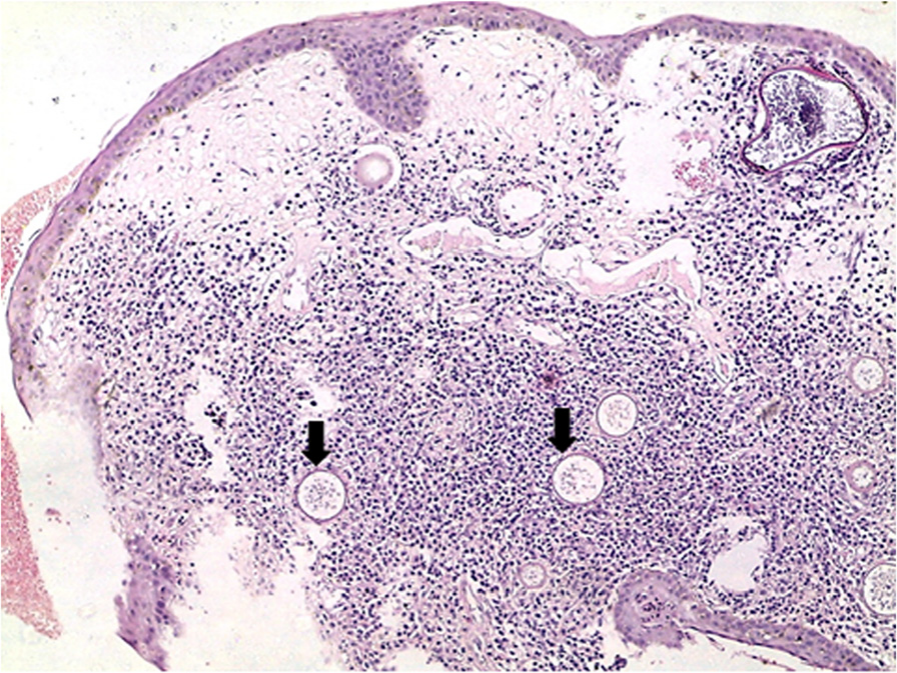
**Photomicrograph of ocular rhinosporidiosis stained with Haematoxylin & Eosin (H & E ×10) showing multiple sporangia within the conjunctival stroma (block arrows).**

**Figure 3:**
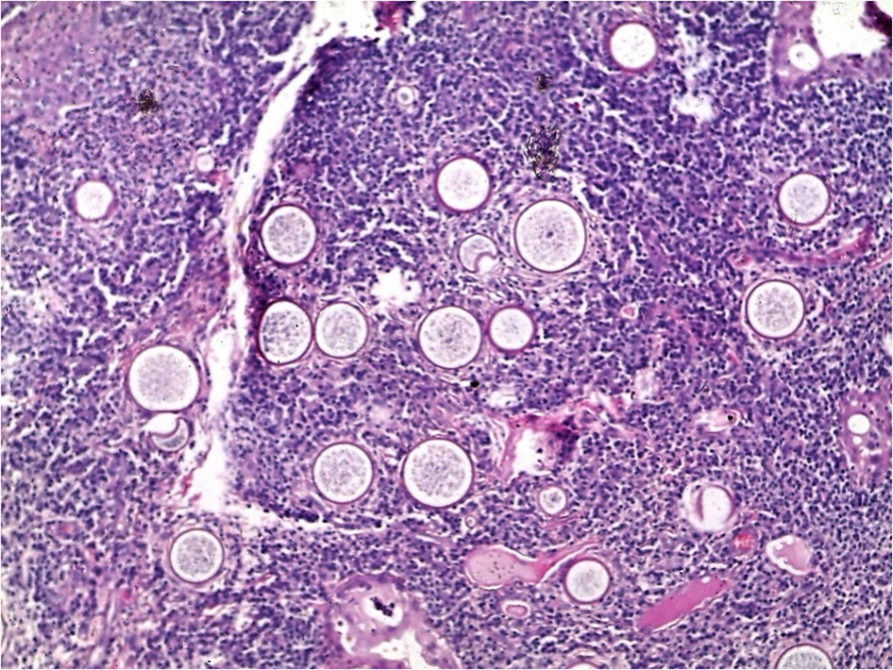
**Multiple sporangia with a reactive mixed inflammatory cell infiltrate (H & E ×20).**

**Figure 4:**
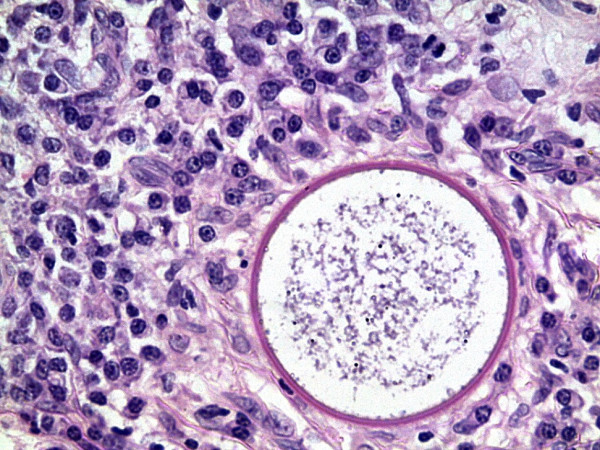
**Sporangium at higher magnification filled with endoconidia and surrounded by plasma cells and lymphocytes (H & E ×40).**

**Figure 5:**
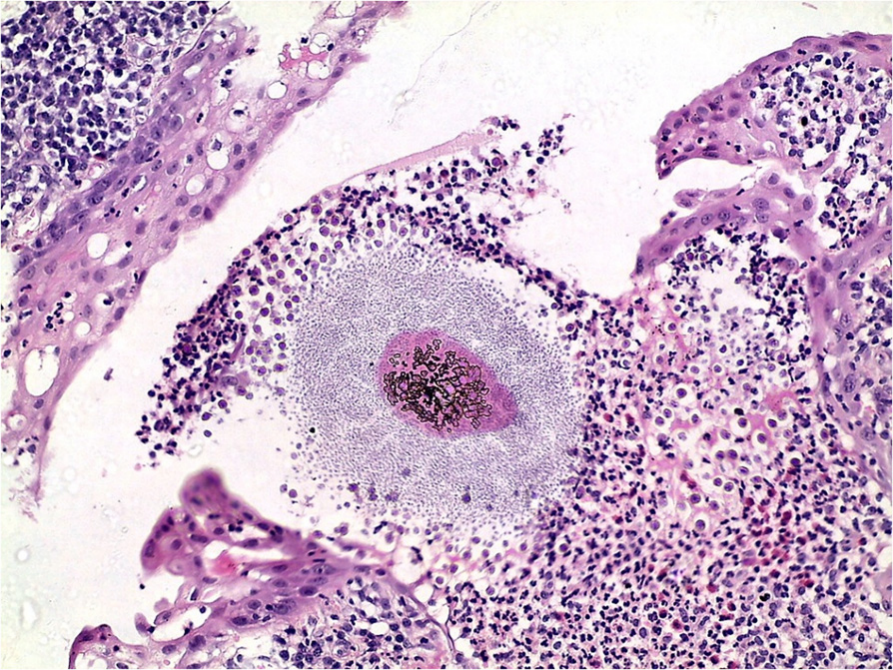
**Burst sporangium with discharged microsporangia surrounded by an inflammatory cell infiltrate (H & E ×40).**

**Figure 6:**
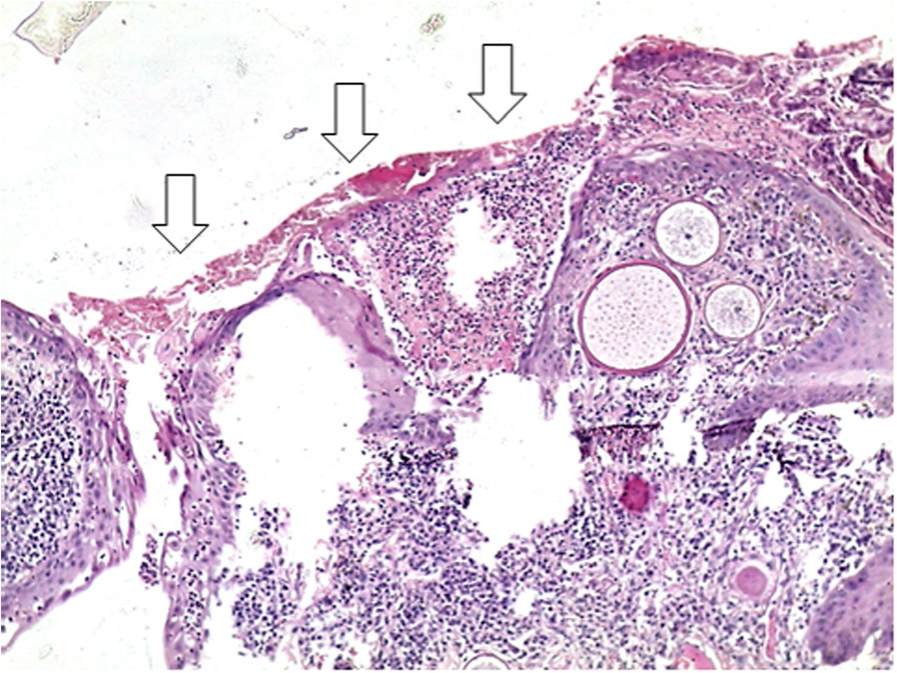
**Ulcerated surface epithelium (open arrows) with a fibrin plaque and granulation tissue on the basal side of the ulcer (H & E ×20).**

Histological analysis revealed multiple sporangia in the conjunctival stroma, an ulcerated squamous epithelium covered by a fibrin plaque whose underlying tissue showed granulomatous tissue, mixed inflammatory cells with lymphocytes showing a maturation spectrum and numerous thick walled sporangia filled with nucleated basophilic endoconidia (Figures 2, 3, 4, 5 and 6). A diagnosis of ocular rhinosporidiosis was made.

**Figure 7:**
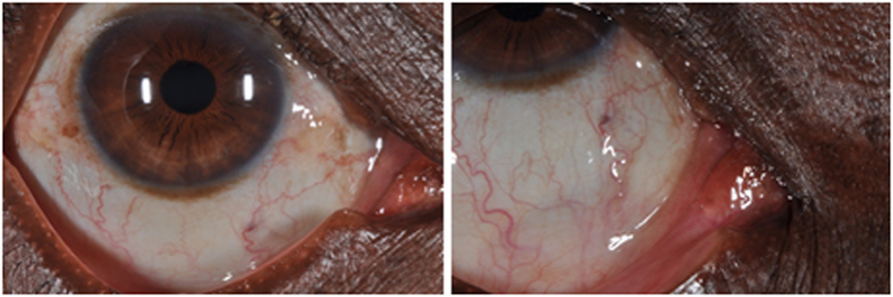
**Post-operative photographs showing no recurrence 6 months after excision of ocular rhinosporidiosis.**

There was no recurrence 6 months after excision was performed (Figure 7).

## Conclusions

Ocular rhinosporidiosis occurs in East Africa. It may resemble conjunctival squamous papilloma. Although toluidine blue has been used as a vital stain of conjunctival lesions, in this case, it was unable to distinguish between an infective and neoplastic cause.

### Consent

Written informed consent was obtained from the patient for publication of this case report and any accompanying images. A copy of the written consent is available for review by the Editor of this journal.

## Competing interests

The authors declare that they have no competing interests.

## Authors’ contributions

SG first evaluated the case, took clinical photographs and conceived the report idea. EM performed the excision surgery. TO performed the histopathological assessment and made the diagnosis. JK reviewed the case on follow up. AMZ coordinated patient and institutional consent for publication of this clinical material. MSS and MJB evaluated the clinical and histopathology photographs. All authors read and approved the final manuscript.

## Pre-publication history

The pre-publication history for this paper can be accessed here:

http://www.biomedcentral.com/1471-2415/14/45/prepub

## References

[B1] Arseculeratne SN Rhinosporidiosis: what is the cause? Curr Opin Infect Dis 2005 18 2 113 118 10.1097/01.qco.0000160898.82115.e8 15735413

[B2] Vilela R Mendoza L The taxonomy and phylogenetics of the human and animal pathogen Rhinosporidium seeberi: a critical review Rev Iberoam Micol 2012 29 4 185 199 10.1016/j.riam.2012.03.012 22504725

[B3] Sudarshan V Goel NK Gahine R Krishnani C Rhinosporidiosis in Raipur, Chhattisgarh: a report of 462 cases Indian J Pathol Microbiol 2007 50 4 718 721 18306535

[B4] Makannavar JH Chavan SS Rhinosporidiosis–a clinicopathological study of 34 cases Indian J Pathol Microbiol 2001 44 1 17 21 12561989

[B5] Mallick AA Majhi TK Pal DK Rhinosporidiosis affecting multiple parts of the body Trop Dr 2012 42 3 174 175 10.1258/td.2012.120035 22785545

[B6] Duke-Elder S Diseases of the Outer Eye 1965 III St. Louis: Mosby

[B7] Salazar Campos MC Surka J Garcia Jardon M Bustamante N Ocular rhinosporidiosis S Afr Med J 2005 95 12 950 952 16465355

[B8] Pe’er J Gnessin H Levinger S Averbukh E Levy Y Polacheck I Conjunctival oculosporidiosis in east Africa caused by Rhinosporidium seeberi Arch Pathol Lab Med 1996 120 9 854 858 9140291

[B9] Venkataramaiah NR van Raalte JA Shabe JK Rhinosporidiosis in Tanzania Trop Geogr Med 1981 33 2 185 187 7281217

[B10] Kaimbo KW Parys-Van Ginderdeuren R Conjunctival rhinosporidiosis: a case report from a Congolese patient Bull Soc Belge Ophtalmol 2008 309–310 19 22 19198547

[B11] Dago-Akribi A Ette M Diomande MI Kioffi Y Honde M D’Ordock AF Beaumel A Clinico-pathologic aspects of rhinosporidiosis in the Ivory Coast. Report of 9 cases observed over 18 years Ann Pathol 1993 13 2 97 99 8395846

[B12] Naik KG Shukla SM Rhinosporidiosis in Zambia Med J Zambia 1980 14 5 78 80 7053007

[B13] Ravisse P Le Gonidec G Moliva B [Report on first 2 cases of rhinosporidiosis in Cameroon] Bulletin de la Societe de pathologie exotique et de ses filiales 1976 69 3 222 224 1037384

[B14] Sood N Agarwal MC Gugnani HC Ocular rhinosporidiosis: a case report from Delhi J Infect Dev Ctries 2012 6 11 825 827 23277509 10.3855/jidc.2397

[B15] Arseculeratne SN Recent advances in rhinosporidiosis and Rhinosporidium seeberi Indian J Med Microbiol 2002 20 3 119 131 17657050

[B16] Belliveau MJ Strube YN Dexter DF Kratky V Bloody tears from lacrimal sac rhinosporidiosis Can J Ophthalmol Can J Ophthalmol 2012 47 5 e23 e24 10.1016/j.jcjo.2012.03.019 23036556

[B17] Ghosh AK De Sarkar A Bhaduri G Datta A Das A Bandyopadhyay A Ocular rhinosporidiosis J Indian Med Assoc 2004 102 12 732 764 15871364

[B18] Varshney S Bist SS Gupta P Gupta N Bhatia R Lacrimal sac diverticulum due to Rhinosporidiosis Indian j otolaryngol head and neck surgery: official publication of the Assoc Otolaryngologists of India 2007 59 4 353 356 10.1007/s12070-007-0100-8 PMC3452259 23120472

[B19] Mukhopadhyay S Shome S Bar PK Chakrabarti A Mazumdar S De A Sadhukhan K Bala B Ocular rhinosporidiosis presenting as recurrent chalazion Int Ophthalmol 2012 1 3 PMID 22986579 10.1007/s10792-012-9625-2 22986579

[B20] Lavaju P Arya SK Kumar B Upadhaya P Conjunctival rhinosporidiosis presenting as a cystic mass-an unusual presentation Nepal J Ophthalmol: NEPJOPH 2010 2 2 157 159 10.3126/nepjoph.v2i2.3724 21505534

[B21] Suh LH Barron J Dubovy SR Gaunt ML Ledee DR Miller D Fell JW Forster RK Ocular rhinosporidiosis presenting as chronic follicular conjunctivitis in a contact lens wearer Arch Ophthalmol 2009 127 8 1076 1077 10.1001/archophthalmol.2009.179 19667358

[B22] Bhomaj S Das JC Chaudhuri Z Bansal RL Sharma P Rhinosporidiosis and peripheral keratitis Ophthalmic Surg Lasers 2001 32 4 338 340 11475404

[B23] De Doncker RM de Keizer RJ Oosterhuis JA Maes A Scleral melting in a patient with conjunctival rhinosporidiosis Br J Ophthalmol 1990 74 10 635 637 10.1136/bjo.74.10.635 2285690 PMC1042237

[B24] Talukder AK Rahman MA Islam MN Chowdhury MH Ciliary staphyloma: very rare sequelae of conjunctival rhinosporiodosis Mymensingh Med J: MMJ 2004 13 1 86 87 14747794

[B25] Sharma KD Shrivastav JB Agarwal S Ocular rhinosporidiosis simulating a tumour Br J Ophthalmol 1958 42 9 572 574 10.1136/bjo.42.9.572 13572774 PMC509704

[B26] Vallarelli AF Rosa SP Souza EM Rhinosporidiosis: cutaneous manifestation An Bras Dermatol 2011 86 4 795 796 10.1590/S0365-05962011000400029 21987153

[B27] Mandal SK Bhakta A Mandal A Biswas BK Giant mass conjunctival rhinosporidiosis causing severe mechanical ectropion J Indian Med Assoc 2012 110 5 328 329 23360029

[B28] Sinha A Phukan JP Bandyopadhyay G Sengupta S Bose K Mondal RK Choudhuri MK Clinicopathological study of rhinosporidiosis with special reference to cytodiagnosis J cytol/Indian Acad Cytologists 2012 29 4 246 249 10.4103/0970-9371.103943 PMC3543593 23326028

[B29] Romero IL Barros Jde N Martins MC Ballalai PL The use of 1% toluidine blue eye drops in the diagnosis of ocular surface squamous neoplasia Cornea 2013 32 1 36 39 10.1097/ICO.0b013e318243f61f 22525782

[B30] Mithal C Agarwal P Mithal N Ocular and adnexal rhinosporidiosis : the clinical profile and treatment outcomes in a tertiary eye care centre Nepal J Ophthalmol 2012 4 1 45 48 22343995 10.3126/nepjoph.v4i1.5849

[B31] Jacob P Rose JS Hoshing A Chacko G Tectonic corneal graft for conjunctival rhinosporidiosis with scleral melt Indian J Ophthalmol 2011 59 3 251 253 10.4103/0301-4738.81046 21586855 PMC3120253

[B32] Arseculeratne SN Chemotherapy of Rhinosporidiosis: a Review J Infect Dis Antimicrob Agents 2009 26 1 21 27

[B33] Arseculeratne SN Atapattu DN Balasooriya P Fernando R The effects of biocides (antiseptics and disinfectants) on the endospores of Rhinosporidium seeberi Indian J Med Microbiol 2006 24 2 85 91 10.4103/0255-0857.25176 16687856

[B34] Arseculeratne SN Atapattu DN Eriyagama NB Human anti-rhinosporidial antibody does not cause metabolic inactivation or morphological damage in endospores of Rhinosporidium seeberi, in vitro Indian J Med Microbiol 2005 23 1 14 19 10.4103/0255-0857.13866 15928415

